# Efficacy of anti-diarrheal activity of *Pedalium murex* L., in wistar albino rats

**DOI:** 10.1186/1471-2334-12-S1-P90

**Published:** 2012-05-04

**Authors:** R Ravikumar, Anusha Baskar, V Nithya, R Haripriya, V Parkavi

**Affiliations:** 1 Department of Biotechnology, Srimad Andavan Arts and Science College, Bharathidasan University, Trichy - 620005, Tamil Nadu, India; 2 Department of Biotechnology, PRIST University, Vallam, Thanjavur, Tamil Nadu, India

## Background

Diarrhea is a major health problem especially for children under the age of 5 and up to 17% of children admitted in the pediatric ward die of diarrhea. A range of medicinal plants with anti-diarrheal properties is widely used by traditional healers.

## Methods

The ethyl acetate extract of *Pedalium murex* L., (250, 500, and 1000 mg/kg body weight) was administered orally to three groups of rats (five animals per group) in order to evaluate the activity of the extract against castor oil-induced diarrhea model in rat. Two other groups received normal saline (5mg/kg) and loperamide (5mg/kg) as positive control. The effect of the extract on intestinal transit and castor oil-induced intestinal fluid accumulation (enteropooling) was assessed.

## Results

At oral doses of 250, 500, and 1000 mg/kg body weight, the plant extract showed pronounced and dose-dependent anti-diarrheal activity. The protective role of the extract at 1000 mg/kg was comparable to that of the reference drug, loperamide (5mg/kg). The extract (1000 mg/kg) produced a decrease in intestinal transit comparable to atropine (5mg/kg), and significantly (p<0.01) inhibited castor oil-induced enteropooling. No mortality and visible signs of general weakness were observed in the rats following the extract administration of up to a dose of 6000 mg/kg.

## Conclusion

The results showed that the extract of *Pedalium murex* L., has a significant anti-diarrheal activity which supports its use in traditional herbal medicine practice.

